# A Systematic Investigation on Complement Pathway Activation in Diabetic Retinopathy

**DOI:** 10.3389/fimmu.2020.00154

**Published:** 2020-02-11

**Authors:** Shahna Shahulhameed, Sushma Vishwakarma, Jay Chhablani, Mudit Tyagi, Rajeev R. Pappuru, Saumya Jakati, Subhabrata Chakrabarti, Inderjeet Kaur

**Affiliations:** ^1^Prof Brien Holden Eye Research Centre, LV Prasad Eye Institute, Hyderabad, India; ^2^Smt. Kanuri Santhamma Center for Vitreo Retinal Diseases, LV Prasad Eye Institute, Hyderabad, India; ^3^Medical Retina and Vitreoretinal Surgery, University of Pittsburgh School of Medicine, Pittsburgh, PA, United States; ^4^Ophthalmic Pathology Laboratory, LV Prasad Eye Institute, Hyderabad, India

**Keywords:** retina, diabetic retinopathy, complement pathway, inflammation, microglia, angiogenesis, vitreous humor

## Abstract

The complement system plays a crucial role in retinal homeostasis. While the proteomic analysis of ocular tissues in diabetic retinopathy (DR) has shown the deposition of complement proteins, their exact role in the pathogenesis of DR is yet unclear. We performed a detailed investigation of the role of the complement system by evaluating the levels of major complement proteins including C3, C1q, C4b, Complement Factor B (CFB), and Complement Factor H (CFH) and their activated fragments from both the classical and alternative pathways in vitreous humor and serum samples from proliferative DR (PDR) patients and controls. Further, the expressions of complements and several other key pro- and anti-angiogenic genes in the serum and vitreous humor were analyzed in the blood samples of PDR and non-PDR (NPDR) patients along with controls without diabetes. We also assessed the pro-inflammatory cytokines and matrix metalloproteinases in the vitreous humor samples. There was a significant increase in C3 and its activated fragment C3bα' (110 kDa) along with a corresponding upregulation of CFH in the vitreous of PDR patients, which confirmed the increased activation of the alternative complement pathway in PDR. Likewise, a significant upregulation of angiogenic genes and downregulation of anti-angiogenic genes was seen in PDR and NPDR cases. Increased MMP9 activity and upregulation of inflammatory markers IL8 and sPECAM with a downregulation of anti-inflammatory marker IL-10 in PDR vitreous indicated the possible involvement of microglia in DR pathogenesis. Further, a significantly high C3 deposition in the capillary wall along with thickening of basement membranes and co-localization of CFH expression with CD11b^+ve^ activated microglial cells in diabetic retina suggested microglia as a source of CFH in diabetic retina. The increased CFH levels could be a feedback mechanism for arresting excessive complement activation in DR eyes. A gradual increase of *CFH* and *CD11b* expression in retina with early to late changes in epiretinal membranes of DR patients indicated a major role for the alternative complement pathway in disease progression.

## Introduction

Diabetic retinopathy (DR), characterized by pathological ocular angiogenesis in retina, is a major cause of irreversible vision loss worldwide, with a global prevalence of 34.6% (1). DR has a complex pathophysiology that encompasses the entire retinal function, including compromised neuronal activity and alterations in retinal vasculature that further lead to gradual neurodegeneration, neuroinflammation, and visible vascular complications (2). The retina, being an immune-privileged organ, has its own unique immune regulatory mechanisms, including retinal neurons and RPE, and immune defense mechanisms comprising the microglial population and the complement system. The retinal immune defense mechanism is alerted by any kind of noxious signal and starts a cascade of inflammatory events as an adaptive response to restore the homeostatic balance (3). Low-level activation of the innate immune mechanisms, specifically the complement system, is required to preserve normal eye homeostasis and maintain retinal integrity while aging (4). However, this protective mechanism can have a detrimental impact if the insults persist for a longer duration and leads to irreversible functional loss, as is seen in neurodegenerative diseases such as Alzheimer's disease, Parkinson's disease, amyotrophic lateral sclerosis (ALS), and age-related macular degeneration (ARMD) (5). Increased complement activation-induced photoreceptor cell death has also been reported in retinal detachment, thereby further highlighting the impact of the complement system in various retinal pathologies (6, 7). The complement system, besides having a major role as an immune defense mechanism, is involved in several tissue-remodeling processes, such as liver regeneration and synaptic pruning during development, and also in retinal angiogenesis and neurodegenerative diseases (8–11). The role of the complement system in angiogenesis is of prime importance, since there are several eye diseases associated with abnormal ocular angiogenesis and neurodegeneration, such as retinopathy of prematurity (ROP), age-related macular degeneration (AMD), and proliferative diabetic retinopathy (PDR) (12–14).

The angiogenic and anti-angiogenic potential of complements in AMD and ROP is quite well-known based on experimental evidence derived from both animal and human studies. Bora et al. in 2005, reported C3 and MAC complex depositions in neovessels in mice model of laser-induced choroidal neovascularization (CNV), while the C3 knock out (C3^−/−^) CNV mice showed an absence of neovascularization, with a reduced level of angiogenic factors, thereby suggesting complement component C3 as a pro-angiogenic factor (15). This was further supported by genetic association studies of complement proteins in AMD pathogenesis, where a strong association of CFB and CFH polymorphism was observed along with a strong deposition of complement components in the RPE-Bruch layer (16–18). Further, the deposition of C3 in the retinal microglia and macrophage population in the outer retinal layers was shown to induce retinal degeneration in a mouse model of AMD (19). A recent study on a retinal ischemic mouse model demonstrated the involvement of the alternative complement pathway, specifically C3 and Factor B, in promoting retinal cell apoptosis and vascular dysfunction (20). Conversely, the anti-angiogenic property of the complement system in a ROP mouse model was also documented, where it was shown that C3 and C5aR were required for inhibiting the polarization of macrophage toward its angiogenic potential (10). We have earlier demonstrated microglia-mediated excessive complement activation in the vitreous of ROP babies compared to age-matched controls, suggesting a possible role of the activation of the alternative complement pathway in ocular angiogenesis (21). This evidence points toward both the protective and the destructive roles of complements in different ocular pathologies that are incumbent on disease-specific changes in the retina while sharing some common clinical features.

Some of the recent clinical studies on DR reported an early neuronal loss in the retina prior to the onset of visible vascular changes (22, 23). Further, many basic research investigations have shown the activation of innate immune cells, mainly the microglial population, at different stages of disease progression in animal and human DR retinal tissues (24, 25). It is also quite evident that microglial activation ameliorates tissue damage with chronic inflammatory response under a prolonged duration of tissue insults (26). Several independent studies done on DR have shown an increased deposition of complement component mediators and effector molecules in the retina and vitreous. These included deposition of C3d and MAC complex in the choriocapillaries of DR eyes and reduced level of glycosylphosphatidyl inositol-anchored inhibitors of complements such as CD55 and CD59 in the walls of retinal vessels of DR eyes, suggesting the role of the complement pathway in DR (27, 28), although it is as yet unclear if this is a cause or an effect of prolonged diabetic insult. Though the vitreous proteome studies have detected several complement proteins such as C3, CFI, CFB, C4A, C4B, C2, C4BPA, CFD, and CFH in PDR subjects (29–32), their expression levels are highly variable among different studies and do not explain their exact involvement in DR pathology as observed earlier in ROP and AMD. Also, the complement genes do not show any genetic association with the risk of DR as observed in AMD and ROP. Lack of suitable tissues for such analysis and variable clinical phenotypes pose a major challenge. Further, since microglia and the complement system are involved in retinal defense mechanisms, it would be important to know whether they act independently or synergistically for contributing to DR pathology and whether these are implicated even in the early stages of DR. We hypothesized that a possible crosstalk between these defense mechanisms in uncontrolled diabetic conditions might ameliorate the development of DR and its progression by inducing neurodegeneration and neuroinflammation in the retina that eventually leads to abnormal angiogenesis, as seen in the later stages of DR. Thus, the present study aimed to understand the possible crosstalk between these defense mechanisms and its role in PDR pathogenesis.

We have initially performed a systematic investigation of the role of the complement pathway in PDR pathogenesis by analyzing classical and alternative pathway complement proteins and their activation in vitreous and serum samples of human DR subjects. In addition, we have also correlated our findings with the expression of complement genes in retinal tissues obtained from diabetic cadaveric donors and blood samples of DR patients and controls. Microglial infiltration and its correlation with inflammation and neovascularization were further evaluated by analyzing angiogenic and inflammatory cytokines in the vitreous. Our study identified a localized elevation of C3, especially the 110 kDa activated fragment C3bα' and a concurrent upregulation of CFH along with activated microglial infiltration in the PDR vitreous. To the best of our knowledge, this is the first report on the upregulation of CFH levels in PDR vitreous as revealed through Western blotting and showed its co-localization with activated microglia, thereby suggesting its involvement in the pathogenesis of DR and possible crosstalk between these two defense systems in PDR progression. Further, a gradual increase in microglial-mediated activation of the alternative complement pathway based on *CFH* and *CD11b* gene expression in early to late changes in DR indicates the clinical relevance of the alternative complement pathway's role as a possible biomarker for disease progression.

## Materials and Methods

### Enrollment of Study Participants and Sample Preparation

The study was performed according to the guidelines of the Declaration of Helsinki and approved by the Institutional Review Board. Vitreous samples (100 μl) were collected from normal controls (*n* = 120) and PDR subjects (*n* = 120) undergoing pars plana vitrectomy with prior written informed consent. Samples were collected in surgery rooms under aseptic conditions and then immediately transferred to the laboratory in cold condition. The samples were then centrifuged at 14,000 rpm for 10 min at 4°C to remove any cellular debris and then stored at −80 degrees for further use. Proteins were lysed in an equal volume of RIPA buffer and precipitated with ice-cold acetone overnight at −80°C. The precipitated proteins were collected by centrifugation at 14,000 rpm for 1 h at 4°C, and the protein pellets were dissolved in 1X PBS containing protease inhibitor. Blood samples were collected in vacutainers from PDR (*n* = 38), NPDR (*n* = 38), and control (*n* = 38) subjects, and the serum was separated within 1 h of sample collection by centrifugation at 1,500 rpm for 15 min. The samples were stored at −80°C and thawed prior to the experiments. The total protein concentration was calculated by bicinchoninic acid (BCA) assay. The demographic details of the subjects from whom the vitreous and serum samples were collected are provided in Tables 1, 2.

**Table 1:** Detailed demographic of study subjects used for vitreous protein analysis.

	**Age**	**Gender**	**Duration of DM**
Control vitreous	55.4 ± 1.02	F, *n* = 60, M, *n* = 40	Nil
PDR vitreous	56.17 ± 0.79	F, *n* = 45, M, *n* =55	15.64 ± 0.83

**Table 2:** Detailed demographics of study subjects used for serum protein analysis and mRNA expression analysis by qPCR.

	**Age**	**Gender**	**Duration of DM**
No-DM	65.8 ± 1.03	F, *n* = 16, M, *n* = 22	Nil
NPDR	59.83 ± 1.32	F, *n* = 14, M, *n* = 24	12.88 ± 1.4
PDR	53.86 ± 1.61	F, *n* = 15, M, *n* = 23	15.05 ± 0.9

### Western Blotting

Western blotting was performed with the vitreous and serum samples to identify the role of the complement pathway in PDR pathogenesis. The levels of total C3 (Ms-C3, Catalog No. sc-28294, Santacruz) and of its activated proteolytic fragments in the vitreous humor were determined under non-reducing conditions in samples collected from PDR and no-DM subjects. Likewise, C3 and its activated fragments in serum samples were compared among PDR, NPDR, and no-DM subjects. The classical complement pathway was evaluated by analyzing proteins such as C1q (Ms C1q, Catalog No. ab71089, Abcam) in serum and C1q and C4b (Ms-C4b, Catalog No. sc-74524, Santacruz) in vitreous. The alternative complement pathway was evaluated by estimating the levels of factor Bb of CFB (Rb CFB, Catalog No. ab 72658, Abcam) and CFH (Ms-CFH, Catalog No. sc-166613, Santacruz). Western blotting for CD11b (Rb CD11b, Catalog No. ab133357, Abcam) was performed in the vitreous samples to evaluate the microglial infiltration under PDR pathogenesis and compared with no-DM control vitreous. SDS-PAGE-separated protein samples were transferred to a PVDF membrane (Catalog No. IPFL00010, Millipore) at a constant voltage of 25 V by wet transfer for a period of 1–2 h. After overnight incubation with primary antibodies at 4°C, IR dye conjugated specific secondary antibodies (Anti -Ms. 680RD, Catalog No. 926–68070, LICOR, Anti -Rb 800CW, Catalog No.926–32211, LI-COR) were added to the blots and incubated for 1 h at room temperature. The details of the antibodies used and their dilutions are given in Supplementary Table S1. The blots were developed, and bands were visualized under a LI-COR image scanner, and the band intensities were quantified and compared between test patients and controls using LI-COR image studio software.

### Periodic Acid-Schiff (PAS) Staining

Cadaveric control (*n* = 3) and diabetic eyes from Type 2 DM with no retinopathy (*n* = 3) were collected in a sterile moist chamber within 24 h of death from Ramayamma International Eye Bank, LV Prasad Eye Institute, Hyderabad, India, according to the Tenets of the Declaration of Helsinki. The retina tissues were removed carefully from the eyes under a dissection microscope and fixed in 10% neutral buffered formalin, and paraffin sections were made. For PAS staining (Catalog No. 375810, Sigma), sections were deparaffinized at 60°C for 20–30 min and hydrated, followed by oxidization with 0.5% Periodic acid solution for 10 min. After washing with distilled water for 5 min, the sections were stained with Schiff's solution (Catalog No. 3952016, Sigma) for 15 min in the dark, followed by washing for 5 min and then staining with Hematoxylin (Catalog No. H3136, Sigma) for 5 min. After washing, final dehydration and clearance was done using alcohol and Xylene (Catalog No. 40575, SD Fine-Chem Ltd) and mounted using DPX (Catalog No. POICHA-R-391780, SD Fine-Chem Ltd). The total number of blood vessels was counted manually in control and diabetic retina, and significance was calculated using a *t*-test.

### Immunohistochemistry (IHC)

For IHC, antigen retrieval was carried out for the deparaffinized tissue sections using pH 6 Tris Citrate buffer. The sections were permeabilized using methanol for 30 min at −20°C, followed by washing thrice with 1X PBS. Blocking was done with 2% BSA, then sections were incubated with primary antibodies overnight at 4°C (Ms C3- Santacruz, 1:50, Catalog No. sc-28294, Ms CFH- Santacruz, 1:50, Catalog No. sc-166613, Rb CXCR4-Santacruz, 1:50, Catalog No. sc- 9036, Rb CD11b-CST, 1:200, Catalog No. 49420, Rb GFAP, 1:300, Dako, Catalog No. Z0334). After washing thrice with 1X PBS, the sections were incubated with appropriate fluorescent labeled secondary antibodies (Goat anti Rb594, Life Tech. Catalog No. A-11012, 1:300, Goat anti-Ms 594, Life Tech. Catalog No. A-11005, 1:300, Goat anti-Rb 488, Life Tech. Catalog No. A-11008, 1:300) for 1 h at room temperature. The sections were counterstained with DAPI, and the staining was examined under a fluorescent microscope (EVOS) using appropriate filters.

### Gelatin Zymography

Gelatin zymography was done to analyze the activity of matrix metalloproteinases (MMP2 and MMP9) in the vitreous samples obtained from PDR and control subjects (33). Briefly, a 10 μg of vitreous proteins from PDR and no-DM controls were mixed with 4X lading dye with a final concentration of 1X and loaded into a gelatin-incorporated SDS-PAGE gel at a constant voltage of 125 V until the dye front reached the bottom of the gel. The gel was washed and kept for incubation with 1X renaturing solution (2.5% v/v Triton X- 100 in d. H_2_O) for 30 min followed by overnight incubation with 1X developing buffer (0.05 M Tris HCl, pH 7.8, 0.2 M NaCl, 5 mM CaCl_2_, 0.02% Brij 35) at 37°C. The gel was stained with Coomassie blue buffered (CBB) solution and de-stained until clear gelatinolytic bands were visible.

### Enzyme-Linked Immunosorbent Assay (ELISA)

ELISA was carried out to evaluate the level of cytokines such as sPECAM, IL-8 and IL-10, sVEGFR1, and VEGF and VEGFR2 in the vitreous samples collected from PDR and control subjects. Vitreous humor samples were diluted with assay buffer at a dilution of 1:3. The standards were prepared by reconstituting them with 250 μL of deionized water. Twenty five micro liter of assay standards and quality controls were added into a 96-well plate, and then 25 μL of diluted test samples (vitreous humor) and 25 μL of targeted antibody-coated magnetic beads were added. The plate was kept for overnight incubation (16–20 h) at 4°C with constant shaking. The plate was then washed thrice with 1X wash buffer followed by incubation with 25 μL of detection antibodies for 1 h at room temperature under dark conditions. Following this, 25 μL of reporter tag comprised of streptavidin-phycoerythrin was added to each of the wells and incubated in the dark with gentle shaking for 30 min. The plates were washed thrice with wash buffer, and 150 μL of sheath fluid was added to each of the wells. The plate was scanned under a Luminex system with xPONENT® software. The generated results were exported in terms of median fluorescent intensity, and the concentrations of the analytes in the PDR and controls were calculated. The significance was calculated based on the *t*-test with a *p* < 0.05 using GraphPad Prism software.

### RNA Isolation and Quantitative Real-Time PCR

Blood samples were collected in K3EDTA-coated 3-mL blood vacutainers from PDR, NPDR, and no-DM subjects, and total RNA was isolated using the TRIzol-chloroform method. RNA was isolated from diabetic (*n* = 7) and control (*n* = 7) retinal tissues and epiretinal membranes collected from PDR (*n* = 4) and control (*n* = 4) subjects while undergoing membrane peeling as a part of their surgical management after written informed consent had been obtained. Then, 1 μg of RNA was converted into cDNA using the iScript cDNA conversion kit (Catalog No. 1708891, Bio-Rad) as per the manufacturer's protocol for RNA obtained from blood and retinal tissues. For membranes, a high-capacity cDNA reverse transcription kit was used (Catalog No. 4368813, Applied Biosystems). Semi-quantitative PCR was performed on a 7900 HT platform using TaqMan assay chemistry for *C3* and *TGF*-β and SyBr green chemistry for the *VEGF, CFH*, and *CXCR4* genes. *C3, CFH*, and *CD11b* expression was also analyzed in retinal tissues and ERM samples from patients and controls using SyBr green chemistry. β-actin was used as the normalization control using standard thermal cycling conditions. The cycle threshold (Ct) values calculated for each test gene were obtained for each sample using SDS2.3 software, and fold change was calculated using 2^−ΔΔCt^. Data are represented as mean ± SEM, and significance was calculated. The primer sequences used for qRT are given in Supplementary Table S2.

## Results

### Demographics of the Study Subjects

Vitreous samples were obtained from the study subjects while undergoing pars plana vitrectomy and blood samples were collected from subjects by venipuncture, with prior informed consent in all cases. None of the patients had received any intraocular anti-VEGF injections as a part of their ocular complications prior to sample collection. Further detailed demographic data for these subjects are provided in Tables 1 and 2.

### Systematic Evaluation of Complement Pathway Activation by Analyzing the Central Complement Protein C3

**Figure 1:**
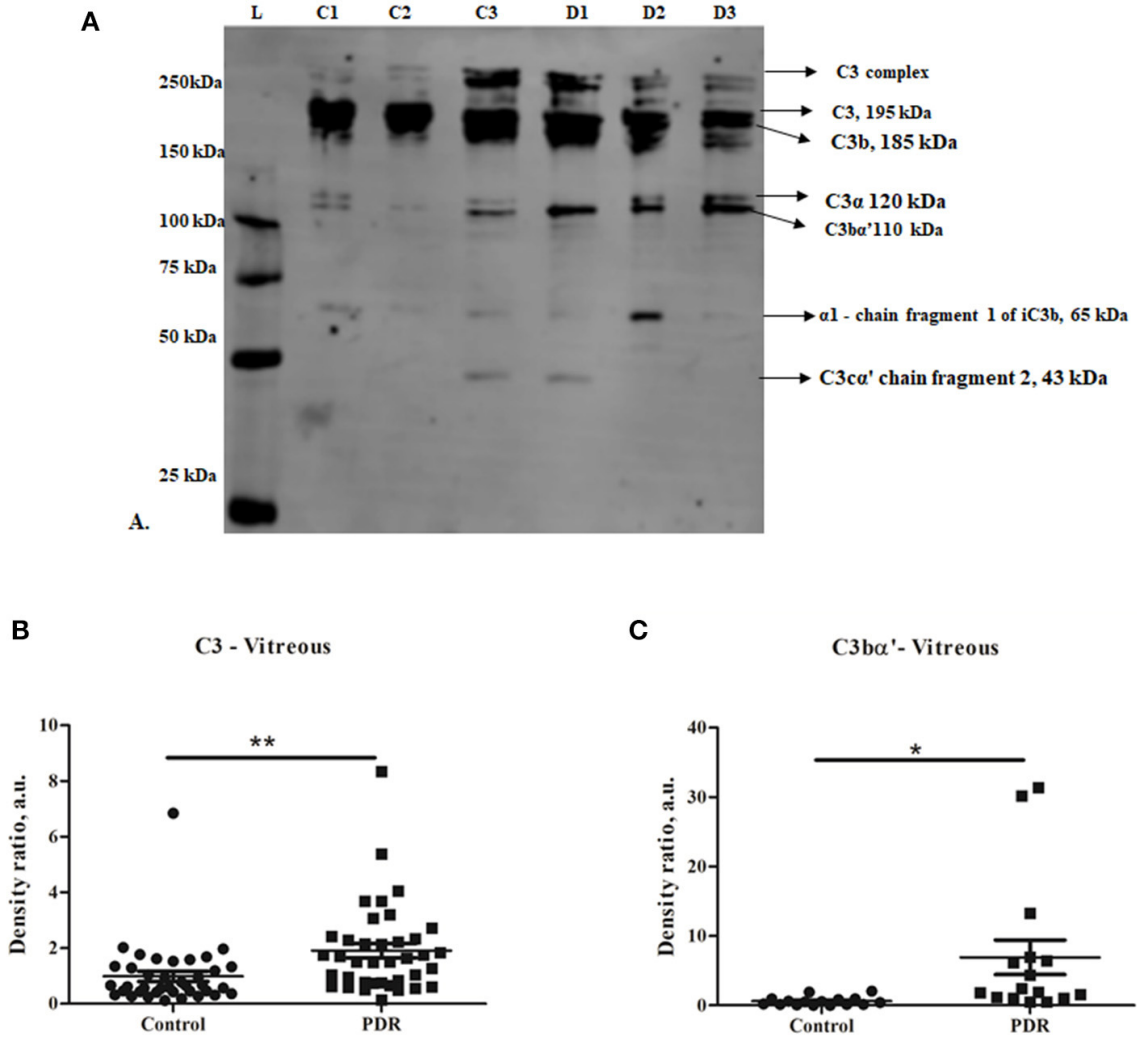
**(A)** Representative Western blot of C3 in PDR and no-DM vitreous. **(B)** Quantification of total C3 in PDR and in no-DM control vitreous by densitometry (PDR, *n* = 38 and Control, *n* = 38). **(C)** Quantification of C3bα' (110 kDa) in PDR (*n* = 16) vitreous compared to control vitreous (*n* = 16). **p = 0.004, *p = 0.03, respectively; data represented as mean ± SEM, C, control vitreous; D, PDR vitreous; L, protein ladder.

Complement component C3 constitutes the central complement proteins that converge all the three pathways of the complement system. In order to understand the complement activation in PDR vitreous, Western blotting for C3 was performed. The levels of total C3 molecules and of individual C3 activated fragments in PDR patients and controls were separated on PAGE and evaluated by quantifying the mean band intensity using Image Studio^TM^ Lite quantification software (LI-COR). These were then compared with no-DM controls based on equal protein loading based on Ponceau staining. There was a significant increase in total C3 in the PDR vitreous (1.9 ± 0.25, ^**^p = 0.004) compared to no-DM controls (0.98 ± 0.18) (Figures 1A,B). The total number of activated C3 fragments, including intact C3 (195 kDa), C3bα' (110 kDa), C3α (120 kDa), C3β (75 kDa), α-1 fragment of iC3b (65 kDa), and C3cα' fragment-2, (43 kDa) were analyzed in the PDR and no-DM controls. There was a significant increase in C3bα' (110 kDa) fragments (^*^p = 0.01) in the vitreous of PDR subjects (6.9 ± 2.47) compared to controls (0.604 ± 0.15) (Figure 1C). Western blotting of the serum samples identified a slight but statistically insignificant increase in total C3 in PDR and NPDR compared to the no-DM samples (PDR vs. no-DM: 1.69 ± 0.58, *p* = 0.7, NPDR vs. no-DM: 1.38 ± 0.24, *p* = 0.7 and PDR vs. NPDR: 1.19 ± 0.23, *p* = 0.9). We did not identify any significant changes for any of the other C3 fragments in the serum samples (Supplementary Figure 1).

### Contribution of the Classical and Alternative Pathway of Complement Activation in Diabetic Retinopathy

**Figure 2:**
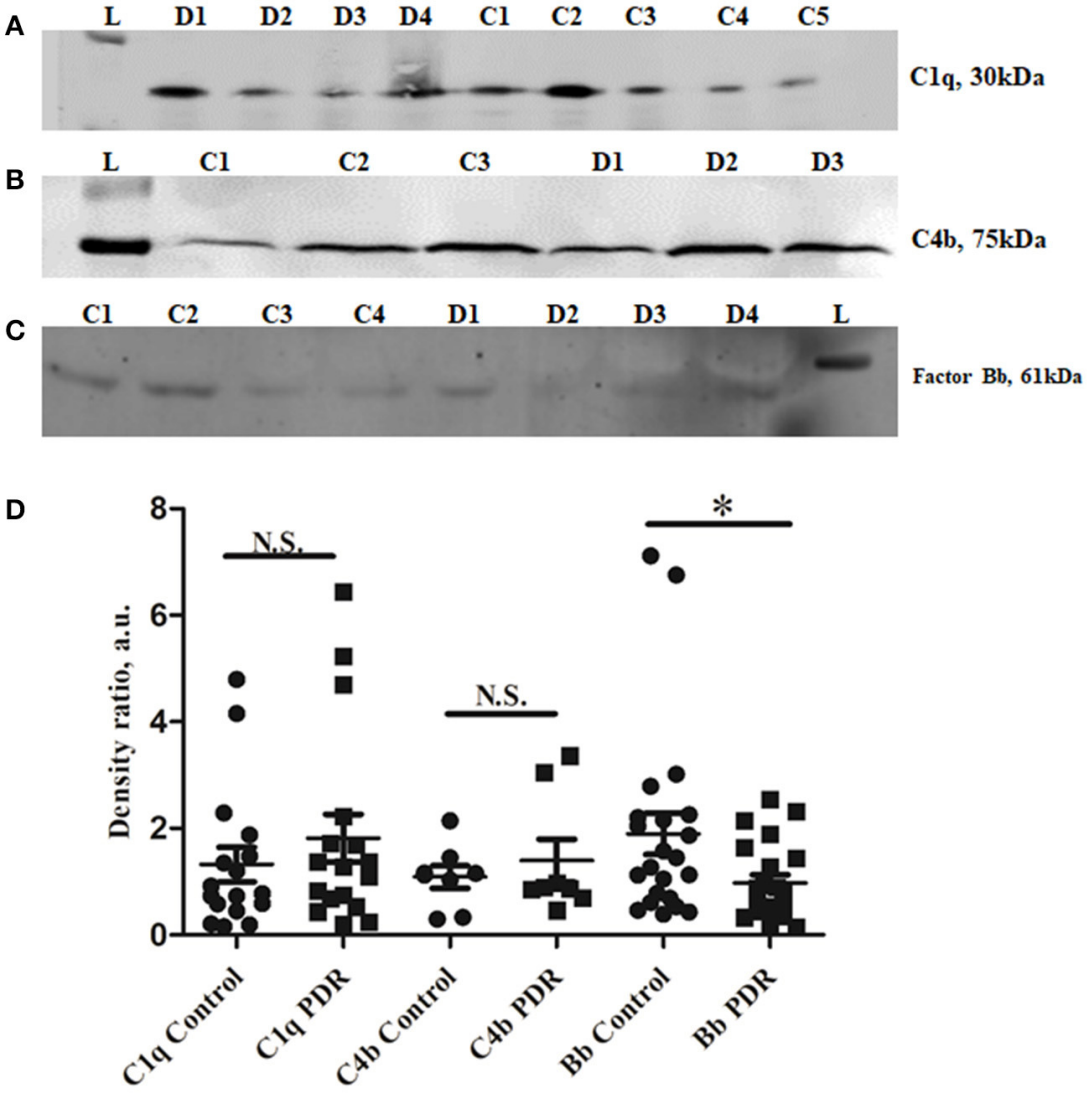
Representative Western blots of **(A)** C1q, **(B)** C4b, and **(C)** Factor Bb in PDR and No-DM controls, **(D)**. Quantification of C1q in PDR (*n* = 17) and no-DM control (*n* = 17) vitreous, *p* > 0.05, C4b in PDR (*n* = 8) and no-DM control (*n* = 8) vitreous, *p* > 0.05 and Bb in PDR (*n* = 22) and no-DM control (*n* = 22) vitreous, *p = 0.03. Data represented as mean ± SEM, D, PDR; C, controls; L, protein ladder; n.s., not significant.

The classical pathway of complement activation was evaluated based on the levels of proteins such as C1q (vitreous and serum) and C4b (vitreous), while the alternative pathway of complement activation was evaluated based on the levels of activated Bb fragment of Factor B (vitreous). The Western blotting results identified no significant change in C1q and C4b in the vitreous of PDR compared to no-DM controls (C1q–PDR: 1.81 ± 0.44, controls: 1.32 ± 0.38 *p* = 0.3; C4b–PDR: 1.39 ± 0.4, control 1.08 ± 0.2, *p* = 0.5) (Figures 2A,B,D). Likewise, there was no significant change in C1q levels in the systemic circulation of PDR and NPDR compared to no-DM controls (PDR vs. no-DM: 1.12 ± 0.43, *p* = *0.5*, PDR vs. NPDR: 0.75 ± 0.54, *p* = 0.5, NPDR vs. no DM: 1.43 ± 0.46, *p* = 0.8) (Supplementary Figures 2A,B). Thus, the classical pathway of complement activation was not involved in the complement activation of DR. However, Western blotting of Bb indicated a 61 kDa band in the vitreous samples (Figure 2C). The mean intensity of the 61 kDa band showed a significant downregulation of Bb in the vitreous of PDR samples compared to the controls (PDR: 0.97 ± 0.15, Controls: 1.89 ± 0.38, ^*^p = *0.03*) (Figure 2E), suggesting that factor B in the PDR vitreous might be bound to form C3 convertase (C3bBb) for the activation of the alternative complement pathway.

### Assessment of Regulation of the Alternative Complement Pathway by Complement Factor H (CFH)

**Figure 3:**
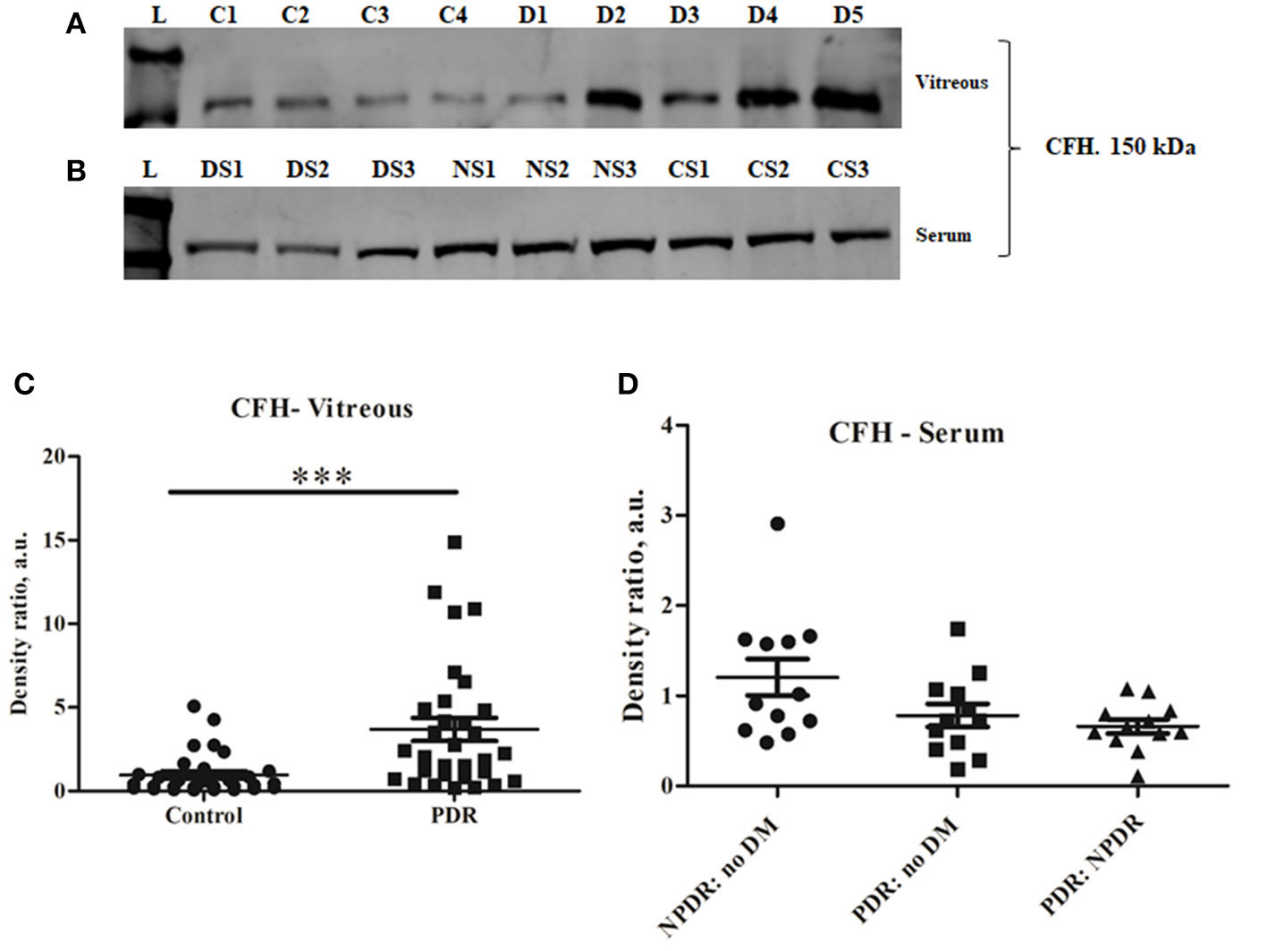
Representative Western blot of **(A)** CFH (150 kDa) in PDR and No-DM controls and **(B)** CFH (150 kDa) in PDR, NPDR, and no-DM control serum. Quantitative estimates of **(C)** CFH band in PDR (*n* = 31) vs. no-DM control (*n* = 31) vitreous, ***p < 0.0004 and **(D)** CFH band in PDR (*n* = 12), NPDR (*n* = 12), and no-DM control (*n* = 12) serum, *p* > 0.05 (not significant). Data represented as mean ± SEM, D, PDR vitreous; C, control vitreous; L, protein ladder; DS, PDR serum; NS, NPDR; CS, Control serum; n.s., not significant.

CFH is a negative regulator of the alternative pathway of the complement. Western blotting of CFH identified a sharp 150 kDa band of CFH, and its levels were significantly higher in PDR compared to control vitreous (controls: 0.96 ± 0.172, PDR: 3.68 ± 0.66, ^***^p = *0.0004*) (Figures 3A,C). In order to identify whether serum infiltration contributed to their increased level in PDR vitreous, CFH levels were independently compared between the serum samples of no-DM, NPDR, and PDR subjects (Figure 3B). CFH was found to be downregulated in the PDR serum as compared to NPDR and controls (PDR vs. no-DM: 0.78 ± 0.12, *p* = 0.2, PDR vs. NPDR: 0.66 ± 0.07, *p* = 0.1, NPDR vs. no-DM: 1.205 ± 0.2, *p* = 0.9) (Figure 3D). This indicated that the increased level of CFH in PDR vitreous was not due to the serum infiltration but was a localized phenomenon in the retina.

### Validation of Complement Activation and CFH Upregulation by Immunohistochemistry Using Diabetic and Non-diabetic Cadaveric Retinal Tissues

**Figure 4:**
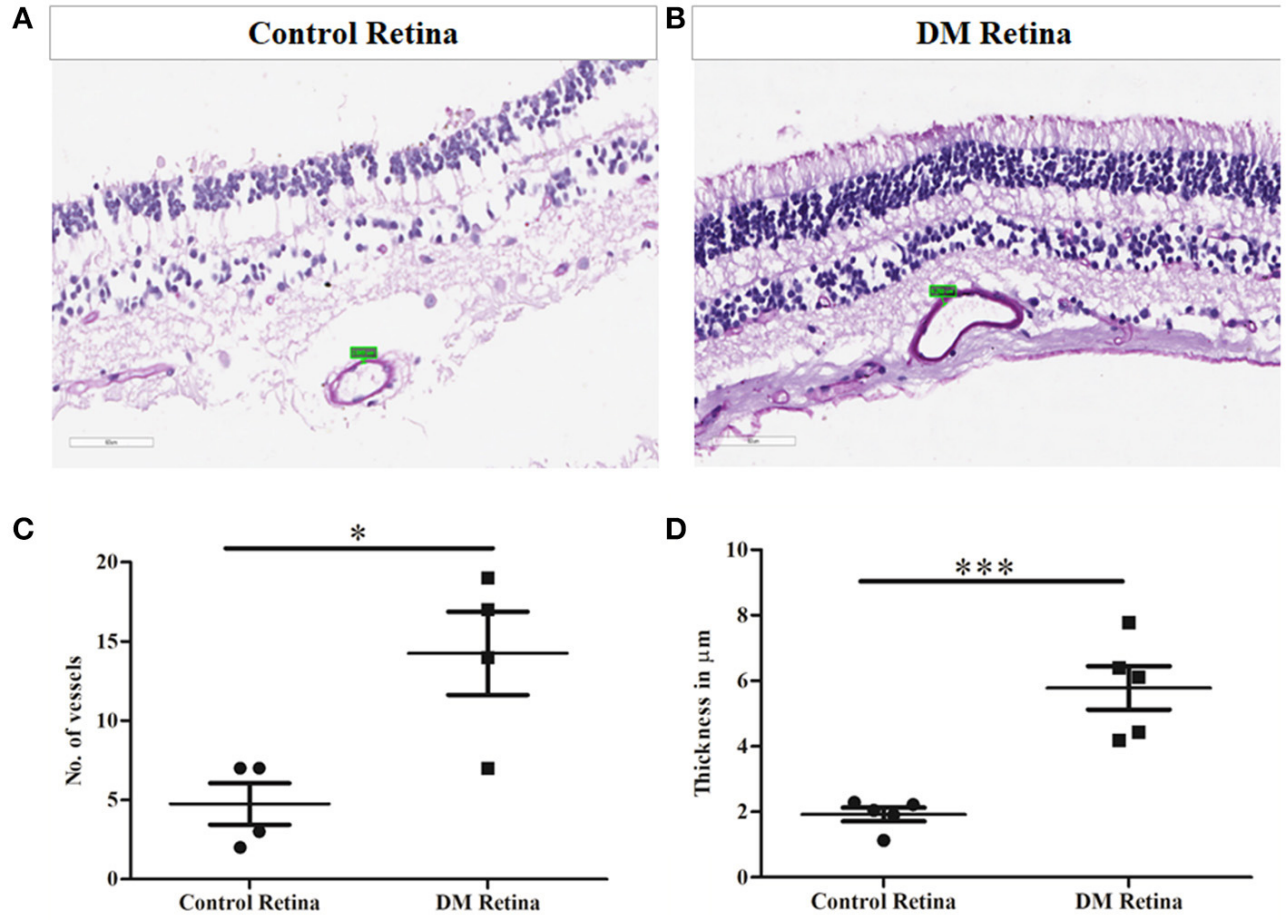
Representative photomicrograph showing thickened and dilated blood vessels in diabetic retina **(B)** in comparison to control retina **(A)** (Periodic acid-Schiff, Magnification: 40X). **(C)** Quantification of number of capillaries in diabetic vs. control retinal tissues. **(D)** Quantification of capillary thickness in diabetic retina vs. control retina. *p = 0.01, ***p = 0.0006.

Prior to the validation of complement activation in retinal tissues, H&E and PAS staining was performed to detect and confirm the early Vascular/DR changes in retina due to a prolonged history of diabetes (Figures 4A,B). The number of choriocapillaries was counted manually in each diabetic and control sections. Mean number of blood vessels was measured and the graph was plotted. A significantly increased number of choriocapillaries was found in diabetic retina compared to control retina (DM: 14.25 ± 3.7, control: 4.7 ± 1.85, ^*^p = 0.01) (Figure 4C). Further, retinal sections were stained using PAS stain. The thickness of vessel walls in five high power fields each in different diabetic retina and control retina were measured using an Aperio Image Scope (version: 12.4.3.5008) at Aperio AT2 Digital Scanner. Mean ± SE was calculated of the thickness in diabetic retina and control retina. A significant increase in vessel wall thickness was observed in diabetic retina compared to control retina (Control: 1.91 ± 0.46, Diabetic retina: 5.78 ± 1.48, ^***^p = 0.0006) (Figure 4D).

**Figure 5:**
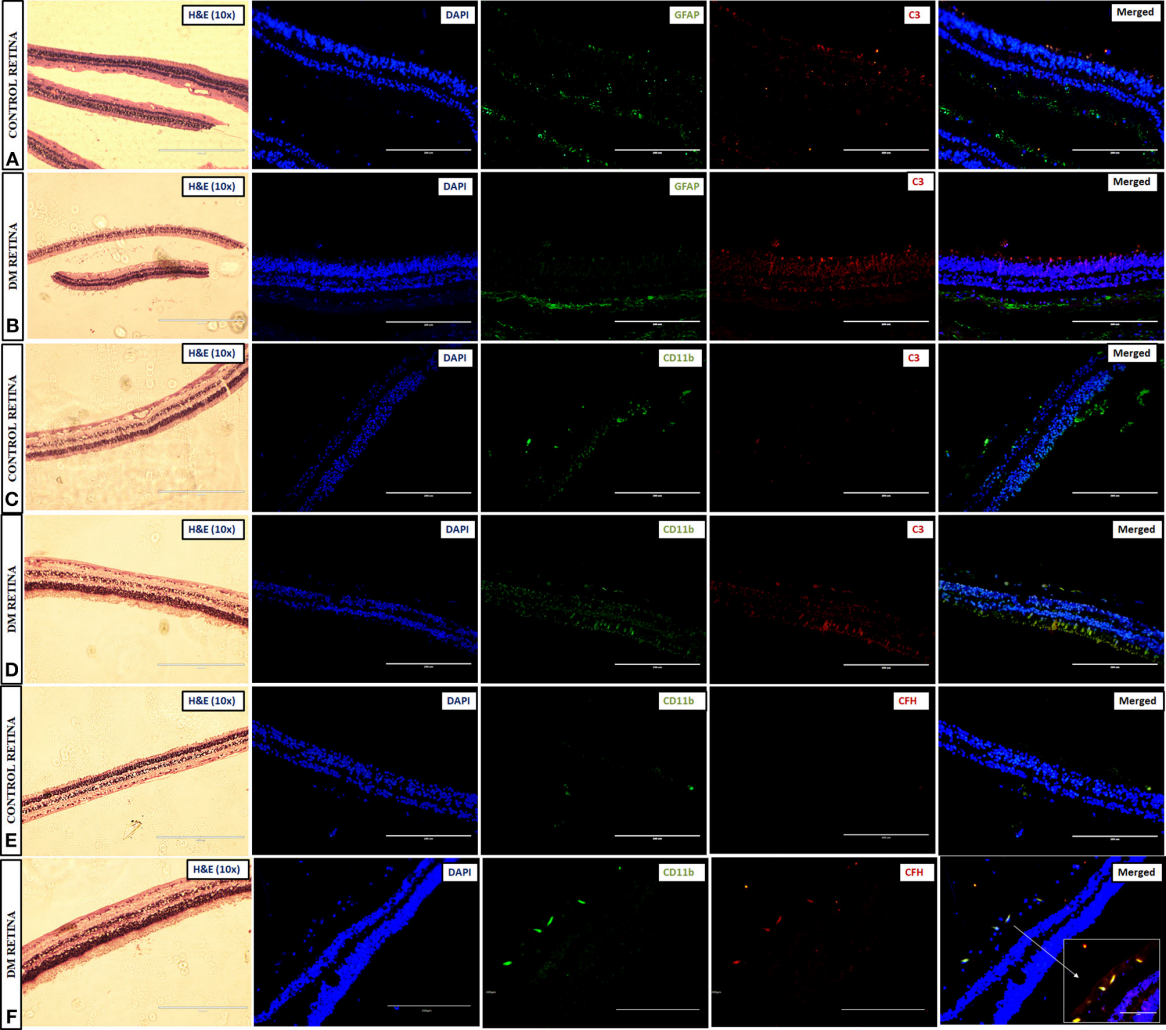
Representative images of the 10X magnified H and E sections and post-immunofluorescence showing the localization of C3 and GFAP in retinal tissues collected from **(A)** control and **(B)** diabetic retina, C3, and CD11b in **(C)** control and **(D)** diabetic retina, and CFH and CD11b in **(E)** control and **(F)** diabetic retina, magnification 20X, scale bar 200 μm. Co-localized expression of CFH and CD11b is highlighted in 40X magnification in panel **(F)**.

Immunohistochemistry (IHC) was performed to test for complement activation and Factor H upregulation in the retina from tissues of diabetic and non-diabetic donor eyes. The retinal tissues were also stained with markers of glial activation such as CD11b for activated microglia and glial fibrillary acidic protein (GFAP) for the macroglial population of the retina. The levels of CXCR4 were also evaluated in these retinal tissues for evaluating the presence of chemotactic cells. The IHC results clearly demonstrated intense staining of C3 in all the retinal layers compared to the control retinas (Figures 5A–D). GFAP was seen only in the inner retinal layers of both DM and control retinas, but the expression of GFAP in diabetic retina was found to be slightly higher than that of control retinas (Figures 5A,B), suggesting the onset of gliosis in DM retinas. Increased expressions of C3 and CD11b were observed in DM retina, whereas in the control retinas, the expressions of these markers were relatively low (Figures 5C,D). Further evaluation of the levels of CFH in retinas from control and diabetic donors (Figures 5E,F) indicated intense staining of CFH co-localizing with CD11b-positive cells in the retinal layers in DM retina, suggesting a possible feedback mechanism for excess complement activation by the microglial cells.

### Assessment of Microglial Infiltration and Activation in Retina as Well as in PDR Vitreous

**Figure 6:**
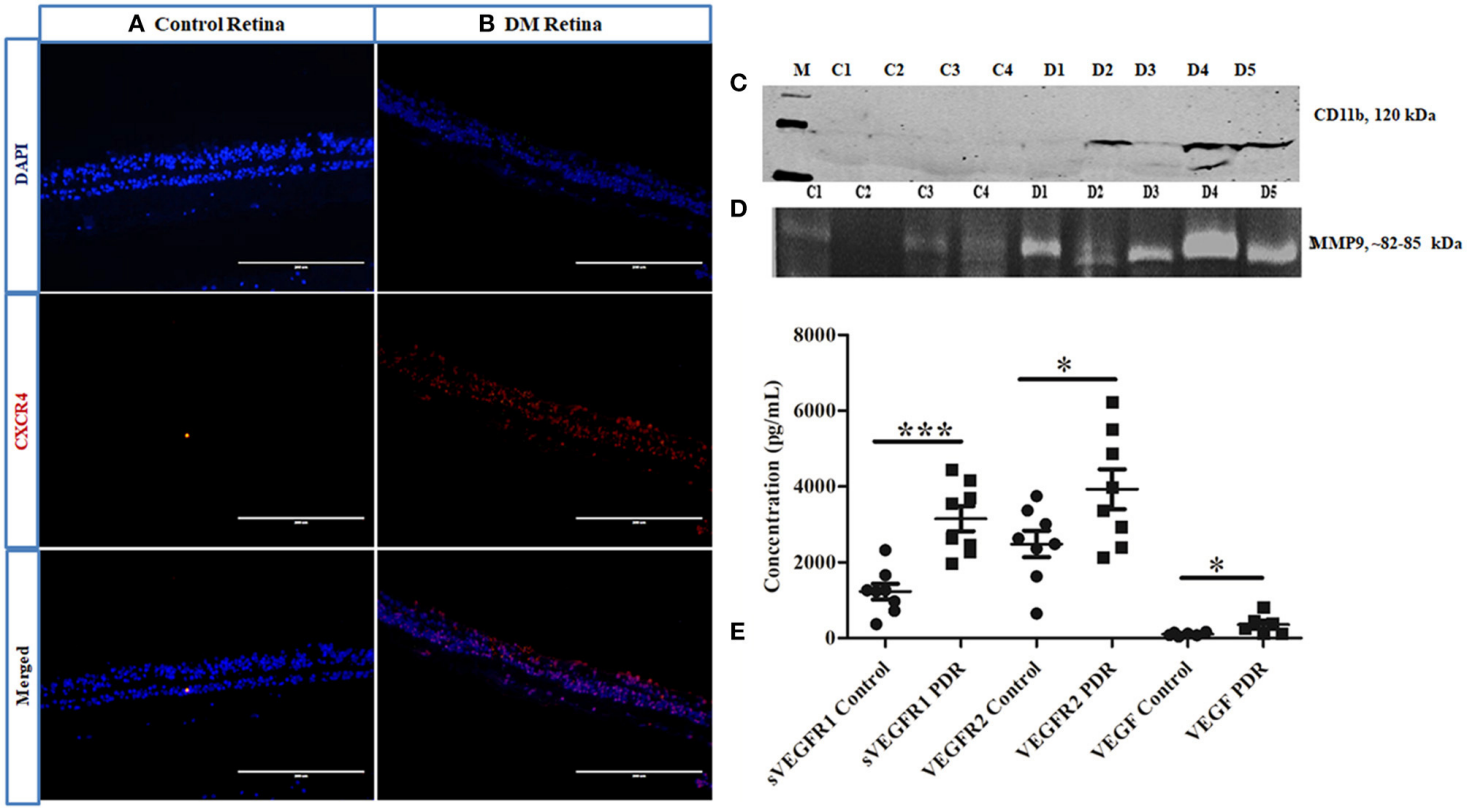
Representative image of **(A,B)** immunofluorescence of CXCR4 in retinal tissues collected from control and diabetic retina, respectively, magnification 20X. **(C)** Representative Western blotting of CD11b in PDR vitreous (*n* = 5) compared to controls (*n* = 4); **(D)** Representative gelatin zymography of vitreous samples from PDR (*n* = 10) vs. controls (*n* = 10) (C, control; D, PDR). **(E)** Scatter plot with individual data points showing differential levels of soluble VEGF (***p = 0.0002) and VEGFR2 (*p = 0.03) (PDR, *n* = 8, Control, *n* = 8) and VEGF (*p = 0.04) (PDR, *n* = 6, Control, *n* = 6) by multiplex ELISA.

Further, the level of CXCR4, a chemokine receptor present in the microglial cells, was evaluated by IHC, which indicated a larger distribution of CXCR4^+ve^cells in DM retina compared to the control retinas, wherein the CXCR4 staining was almost negligible, suggesting microglial activation, and enhanced chemotaxis in diabetes (Figures 6A,B). To evaluate whether this microglial population infiltrated into the vitreous cavity during the advanced stages of the disease, the levels of CD11b were also evaluated in the vitreous samples by Western blotting. The results indicated an intense band of activated microglial marker CD11b in the vitreous of PDR subjects that was absent in the controls (Figure 6C), providing additional evidence of microglial infiltration during PDR pathogenesis. In order to validate the contribution of microglial activation in disease progression, the activity of matrix metalloproteinases was evaluated in PDR vitreous samples. Microglia are the major source of gelatinolytic MMPs such as MMP9 and MMP2 in the retina. Both the levels and enzymatic activity of MMPs in the PDR vitreous were evaluated by gelatin zymography in the vitreous samples of patients and controls. The results indicated a clear gelatinolytic band of 82–85 kDa molecular weight in both PDR and control vitreous that corresponded to active MMP9. However, it was more pronounced in PDR vitreous, indicating increased gelatinolytic activity, and active MMP9 under the disease condition (Figure 6D). Since the upregulation of MMP9 activity is known to drive the growth of blood vessels, the levels of VEGF and VEGFR2 were compared in the vitreous samples of PDR and control subjects. A significant increase in the levels of sVEGFR1, VEGFR2 and VEGF was observed in the vitreous of PDR subjects compared to the controls (sVEGFR1, Control: 1227 ± 209, PDR: 3152 ± 327, ^***^p = 0.0002, VEGFR2, control: 2485 ± 348, PDR: 3929 ± 526, ^*^p = 0.03, VEGF, control: 105 ± 18.12, PDR: 356 ± 106, ^*^p = 0.04) (Figure 6E).

### Quantitative Estimation of Inflammation in PDR and Control Samples Based on the Levels of Pro- and Anti-inflammatory Cytokines

**Figure 7:**
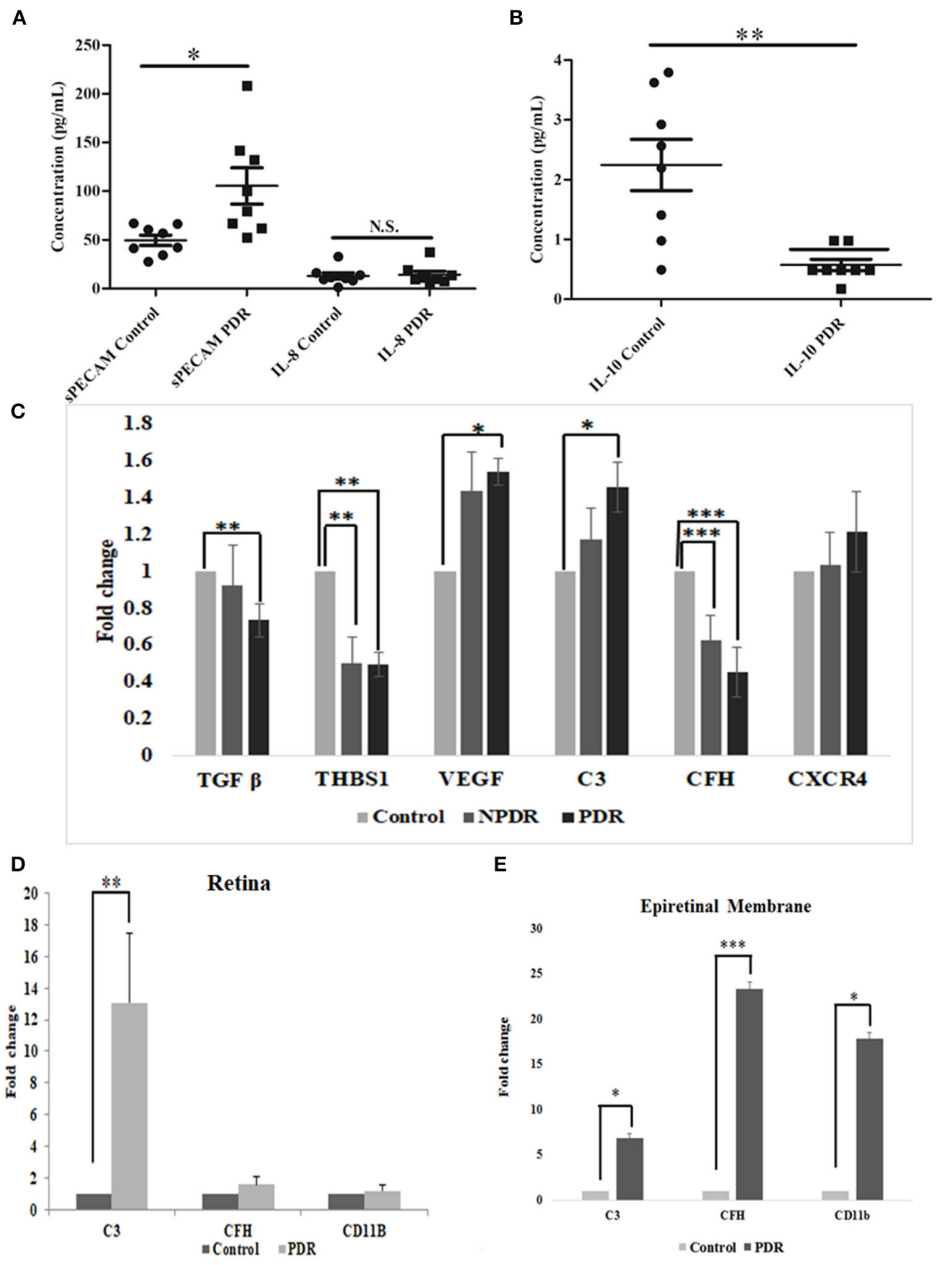
Scatter plot with individual data points showing the quantitative estimation of inflammation in the vitreous samples based on Multiplex ELISA from PDR (*n* = 8) vs. controls (*n* = 8) (C, control; D, PDR) for **(A)** sPECAM and IL-8 and **(B)** IL-10 in PDR vs. control vitreous. Differential expression based on quantitative PCR for **(C)** complement and angiogenic genes from blood in PDR (*n* = 20) and NPDR (*n* = 20) vs. no-DM controls (*n* = 20), **(D)** early (diabetic retina) vs. late changes, and **(E)** ERM tissues in the proteins involved in the activation of the alternative complement pathway. Data represented as mean ± SEM. *p ≤ 0.05, **p ≤ 0.01, ***p ≤ 0.001; ns, *p* > 0.05.

The levels of inflammation in the vitreous samples were analyzed by the quantitative estimation of pro-inflammatory markers such as sPECAM and IL-8, along with an anti-inflammatory marker, IL-10. The levels of sPECAM and IL-8 were found to be higher in the vitreous of PDR compared to the controls (sPECAM: Control: 49.54 ± 4.76, PDR: 105.45 ± 16.69, ^*^p = 0.01, IL-8: Control: 12.79 ± 3.13, PDR: 29.47 ± 14.14, *p* = 0.2). In contrast, the level of anti-inflammatory cytokine IL-10 was found to be significantly downregulated in PDR vitreous as compared to the controls (Control: 2.24 ± 0.42, PDR: 0.57 ± 0.09, ^**^p = 0.001) (Figures 7A,B).

### Gene Expression Profiling for Complement and Angiogenic Genes by Quantitative Real-Time PCR Among Different Disease Categories

Total RNA was isolated from PDR, NPDR, and control subjects, and quantitative real-time PCR was performed for the candidate genes such as *TGF*β*1, THSB1, CXCR4, VEGF, C3*, and *CFH*. Significant changes in the gene expressions were observed for NPDR and PDR compared to controls for *THSB and CFH* (THSB1: NPDR vs. Control: 0.499 ± 0.15; ^**^p = 0.008, PDR vs. Control: 0.495 ± 0.09, ^**^p = 0.004) (CFH: NPDR vs. Control: 0.628 ± 0.12; ^***^p = 0.0009, PDR vs. Control: 0.45 ± 0.05, ^***^p = 0.0001), whereas genes such as *TGF* β*, VEGF*, and *C3* were found to be significantly changed only in PDR compared to controls (PDR vs. Control: *TGF* β: 0.734 ± 0.05, ^**^p = 0.004, *VEGF*: 1.538 ± 0.38, ^*^p = 0.02, *C3*: 1.457 ± 0.56, ^*^p = 0.01), and *CXCR4*- did not show any significant change (Figure 7C).

### Activation of Microglia and CFH in Early vs. Advanced Stages of the Disease

Further, the differential expression profiling of genes involved in complement and microglial activation was performed in eyes with early- (retina from diabetic donor eyes) and late-stage DR (Epiretinal membranes [ERM]) to investigate their role in disease development and progression. Retinal tissues from diabetic donors with early DR changes showed a significant increase for the *C3* gene (13.082 ± 4.24, ^**^p = 0.008) compared to the control retina (without a history of diabetes and vascular changes), while it was insignificant for the *CD11b* (1.2 ± 0.32) and *CFH* (1.56 ± 0.49) genes (Figure 7D). On the contrary, eyes with late-stage DR exhibited significant changes in the expressions of complement genes (*C3:* 6.871 ± 1.85, ^*^p = 0.05 and *CFH: 2*3.35 ± 1.79, ^**^p = 0.007) and *CD11b* (17.88 ± 2.3, ^*^p = 0.01), which is a marker for microglial activation (Figure 7E). It may be noted that changes in the expression of the *CFH* and *CD11b* genes involved in microglial activation were much higher than in the *C3* gene in the ERM tissues of patients with late DR changes.

## Discussion

Diabetic retinopathy is a serious neuro-vascular complication of the retina. The involvement of complement pathway genes in DR progression was proposed based on the identification of complement deposits in choriocapillaries of DR retina and reduced levels of complement pathway inhibitors in diabetic retina, though the mechanism and timing of their involvement were unclear (27, 28). The major aspect of this study was to check whether complement pathway proteins are involved in the early DR pathology or only in DR progression after being released into the vitreous cavity by blood-retina barrier breakdown in the advanced stages based on the analysis of retinal tissue specimens from diabetic and non-diabetic individuals.

C3 is the central complement protein, and the activation of complement pathways causes proteolytic fragmentation of C3. These fragments can bind to the nearby tissues and enhance the inflammatory process (34). Hitherto, a comprehensive study conducted by Garcia et al. identified a significant increase in 42 k Da fragment of C3 in PDR vitreous and found it to correlate with the mRNA expression in diabetic retina (29). In the present study, among the various fragments of C3 protein, a significant upregulation of only the C3bα' (110 kDa) fragment was noted in PDR vitreous. The reactive C3bα' is generated from the 120 kDa α-chain of C3 after the proteolytic removal of the 10 kDa C3a fragment and is part of active C3b (35). A significant increase in the level of C3bα' is indicative of enhanced complement pathway activation in PDR vitreous. However, no significant changes in the expression of the other complement proteins C1q and C4b in PDR and NPDR clearly ruled out any major involvement of the classical complement pathway in the disease pathogenesis.

A detailed analysis of the regulatory proteins involved in the alternative complement pathway such as CFB and CFH indeed confirmed a definitive role of the alternative pathway in DR pathogenesis. CFB, a specific protein, is required for the formation of C3 convertase (C3bBb) for activating the alternative complement pathway (36). The faintly visible but significant downregulation of fragment Bb of factor B seen in PDR vitreous (based on densitometry estimation) could be due to the formation of more C3bBb in PDR vitreous. CFH is a negative regulator/inhibitor of the alternative complement pathway, as it competes with FB for C3b binding and also acts as a co-factor for factor I to degrade C3b to C3bi (37). Typically, a low level of CFH is expected in PDR vitreous concurrent with the downregulation of free Bb in the PDR vitreous. However, an unexpected significant upregulation of CFH could be a feedback mechanism for maintaining the level of C3bα' in PDR vitreous. Several earlier studies have shown that the C3bα' region of C3b protein is involved in binding to CFH protein (38), and this possibly explains the significant upregulation of CFH. This feedback regulation of the alternative complement pathway by CFH was confirmed by a perfect correlation (*r* = 0.78, ^*^p = 0.01) in the increased levels for both CFH and C3bα' in randomly selected vitreous samples of PDR and no-DM controls (Supplementary Figure 3). On the other hand, the downregulation of serum CFH levels in PDR cases compared to NPDR and controls suggests that the upregulation of CFH seen in vitreous is contributed by the local/resident cells in the retina. It was previously reported that VEGF inhibition reduces CFH production in eye and kidney through reduced VEGFR2/PKC-α/CREB signaling (39). Therefore, the observed upregulation of CFH could possibly have been mediated by a simultaneously increased VEGF level in the PDR vitreous. Further, intense staining of complement proteins in the retinal tissues of diabetic donors with early vascular/DR changes as compared to control non-diabetic donor retina confirmed the role of CFH and C3 in diabetic retinopathy. Vascular basement membrane thickening is one of the earliest changes that occur in the retina due to diabetes (40). The thickening of basement membrane, as seen in PAS staining of the studied retinal tissues from the diabetic donor eyes, confirmed the early vascular stage of DR.

The retinal macrophages and microglia are known to synthesize C3 in aging retina (41). However, the present study identified a rather uniform distribution of complement deposition in all of the neural layers of diabetic retina, suggesting thereby that C3 and CFH could also be synthesized by other retinal cell types, including the CD11b^+ve^ microglial population. The CFH in the neural retina was known to have an affinity toward the CR3 receptor in the microglial cells (42). This further indicates that CFH upregulation in PDR could also be related to microglial activation. Surprisingly, increased CFH staining was found in the CD11b^+ve^ microglia in the inner nuclear layers in diabetic retina. This finding was consistent with our earlier report on retinopathy of prematurity (21). Significant gliosis was evidently seen in the diabetic retina tissues, as suggested by the upregulation of GFAP protein, while no expression of complement proteins was seen in the macroglial (Müller glia and astrocytes) cells, further emphasizing a major role of microglia in DR pathogenesis. Microglial cells, being the resident cells, become activated and move up from deep RGC layers toward photoreceptors. Microglial migration upon activation in the diabetic retina was confirmed based on the staining of CXCR4, a chemokine receptor known to be involved in both astroglial activation and microglial signaling (43) and increased levels of activated microglial protein CD11b in vitreous samples on Western blotting. Since an increased level of complement activation is known to cause damage to the retinal tissues (44), it could be speculated that the production of CFH by activated microglia prevents the damage of retinal neurons; this needs to be explored further.

IL-8, a pro-inflammatory cytokine, and IL-10, an anti-inflammatory cytokine, are secreted mainly by M1 and M2 microglia, respectively (45). The downregulation of IL-10 and upregulation of IL-8 further confirmed the activation of the proinflammatory M1 phenotype of microglia in PDR vitreous. Microglia, once activated, are known to secrete the matrix metalloproteinases (46). Together with an increase in pro-inflammatory markers such as IL-8 and MMP9, a significant upregulation of sPECAM and VEGF-VEGFR2 in the PDR vitreous confirmed excessive inflammation and angiogenesis in the PDR eyes. This further suggests that an inflammatory environment in the PDR retina could be a driving force for microglial activation and retinal damage in PDR.

**Figure 8:**
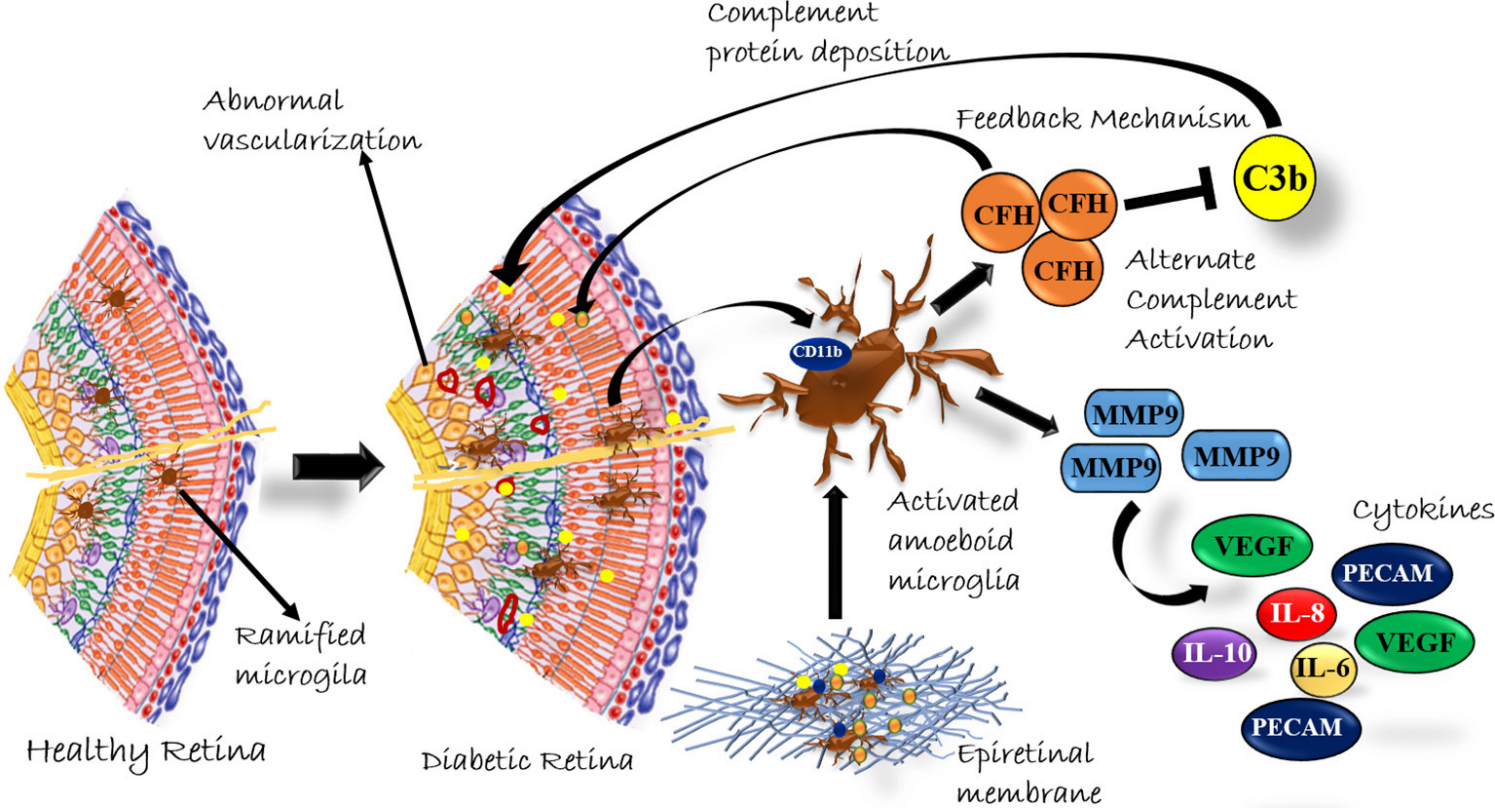
A Schematic summary of the role of complement activation by microglial cells in diabetic retinopathy.

In conclusion, our study provided a systematic analysis of classical and alternative complement pathway activation in PDR pathogenesis. The study, for the first time, showed a significant upregulation of 110 kDa C3bα' and concurrent increase of CFH in PDR vitreous, and this upregulation of complement cascade was localized to retina and not contributed by the blood-retina barrier breakdown that is a common sight in advanced PDR cases. The faintly visible detection of CFB in Western blotting could be due to a low concentration of detectable free CFB protein in the vitreous sample or a poor antibody. The correlation of genetic associations of variations in complement factor H and complement factor B with their expression in epiretinal membrane tissues and vitreous humor could firmly establish the role of the alternative complement pathway in DR pathogenesis. Lastly, our study suggested that the synergistic role of activated microglia and complement activation plays a major role in PDR pathogenesis (Figure 8). In the future, targeting microglial mediated complement activation could pave the way for effective therapeutic management of DR by reducing underlying neuro-inflammation and abnormal angiogenesis.

## Data Availability Statement

The raw data supporting the conclusions of this article will be made available by the authors, without undue reservation, to any qualified researcher.

## Ethics Statement

The studies involving human participants were reviewed and approved by LV Prasad Eye Institute Ethics committee. The patients/participants provided their written informed consent to participate in this study.

## Author Contributions

IK conceived the idea and served as principal investigator. IK, JC, and SC wrote the protocol. SC, JC, MT, RP, SJ, and SC were co-investigators. SS performed most of the work and the protein analysis and IHC of retina and vitreous, SV performed gene expression analysis, IHC, and PAS staining for retina and retinal membranes, and SJ guided the IHC and PAS staining analysis. SS, SV, IK, and SC analyzed the data and wrote the manuscript, and all authors revised the paper and approved the submitted version.

### Conflict of Interest

The authors declare that the research was conducted in the absence of any commercial or financial relationships that could be construed as a potential conflict of interest.
