# Supplementary material for: A Systematic Investigation on Complement Pathway Activation in Diabetic Retinopathy
Source: Front Immunol. 2020 Feb 11;11:154. doi: 10.3389/fimmu.2020.00154 (PMC7026189; doi:10.3389/fimmu.2020.00154)
Supplement: Supplementary file 1 [file Data_Sheet_1.pdf]

Supplementary Materials

Supplementary Table S1: Details of antibodies used for western blotting and their dilutions.

Antibody list	Conc. of protein used	Dilution	% of gel and condition used
Ms C3, Santacruz-sc-28294	15 µg	1:300	7.5% SDS PAGE, non-reducing condition
Ms C1q, Abcam-ab71089	30µg	1:1000	12% SDS PGAE, reducing condition
Ms C4b, Santa Cruz, sc-74524	25µg	1:200	10%SDS PAGE, non-reducing condition
Rb CFB, Abcam, ab72658	25µg	1:500	10%SDS PAGE, non-reducing condition
Ms CFH, Santacruz, sc-166613	30 µg	1:200	7.5%SDS PAGE, reducing condition
Anti -Ms. 680RD, Licor-catalogue no. 926-68070	-	1:75,00	-
Anti -Rb 800CW, Licor, catalogue no.926-32211	--	1:10,000	--

Supplementary Table S2: Primer sequence and TaqMan assays used for qRT PCR

Gene	Forward primer	Reverse primer	Ref.
CXCR4	AGCATGACGGACAAGTACAGG	GATGAAGTCGGGAATAGTCAGC	(Vaday et al., 2004)
Vegf 165	ATCTTCAAGCCATCCTGTGTGC	CAAGGCCCACAGGGATTTTC	(Medford et al., 2009)
CFH	TACTGGCTGGATACCTGCTC	CCTGACGGAGTCTCAAAATG	(Zhang et al., 2015)
β Actin	TCTACAATGAGCTGCGTGTG	GGTGAGGATCTTCATGAGGT	(Horvatinovich et al., 1994)

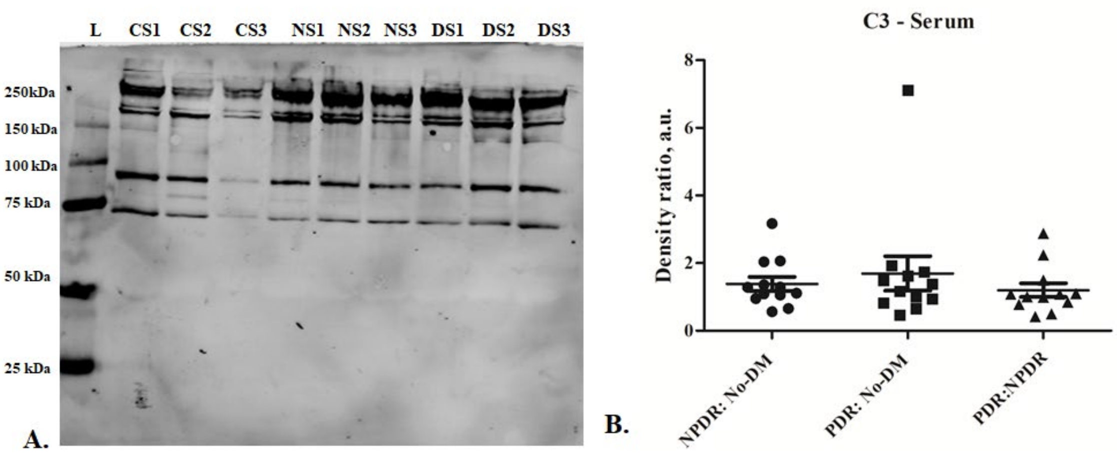

Supplementary Figure 1. (A) Representative Western blot of C3 in serum of PDR, NPDR, and No-DM controls. (B) Quantification of C3 in PDR vs. no-DM (n=12) and no-DM control (n=12); data represented as mean ± SEM. DS: PDR serum, NS: NPDR serum, CS: control serum, L: protein ladder, n.s.: not significant.

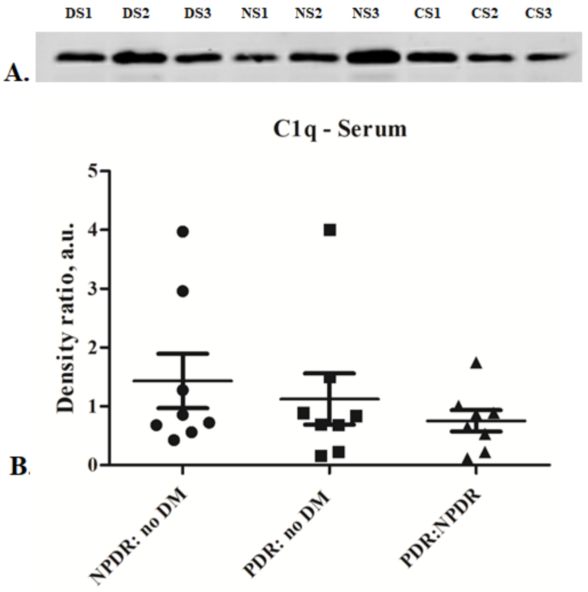

Supplementary Figure 2. (A) Representative Western blot of C1q in serum of PDR, NPDR, and No-DM controls. (B). Quantification of C1q in PDR (n=8) and no-DM control (n=8); data represented as mean ± SEM. DS: PDR serum, NS: NPDR serum, CS: control serum, L: protein ladder, n.s.: not significant.

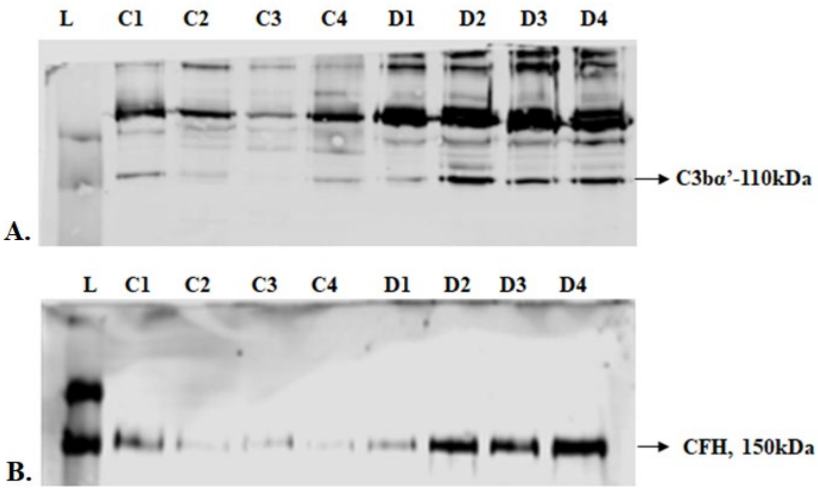

Supplementary Figure 3. Representative Western blot images of (A) C3 and (B) CFH in PDR and no-DM control vitreous. In the Western blots, three of four PDR vitreous samples (D2, D3, and D4) showed higher C3bα' and a concurrent increase in CFH levels.

Horvatinovich, J.M., Sparks, S.D., and Borowitz, M.J. (1994). Detection of terminal deoxynucleotidyl transferase by flow cytometry: a three color method. *Cytometry* 18(4), 228-230. doi: 10.1002/cyto.990180407.

Medford, A.R., Douglas, S.K., Godinho, S.I., Uppington, K.M., Armstrong, L., Gillespie, K.M., et al. (2009). Vascular Endothelial Growth Factor (VEGF) isoform expression and activity in human and murine lung injury. *Respir Res* 10, 27. doi: 10.1186/1465-9921-10-27.

Vaday, G.G., Hua, S.B., Peehl, D.M., Pauling, M.H., Lin, Y.H., Zhu, L., et al. (2004). CXCR4 and CXCL12 (SDF-1) in prostate cancer: inhibitory effects of human single chain Fv antibodies. *Clin Cancer Res* 10(16), 5630-5639. doi: 10.1158/1078-0432.CCR-03-0633.

Zhang, Y., Huang, Q., Tang, M., Zhang, J., and Fan, W. (2015). Complement Factor H Expressed by Retinal Pigment Epithelium Cells Can Suppress Neovascularization of Human Umbilical Vein Endothelial Cells: An in vitro Study. *PLoS One* 10(6), e0129945. doi: 10.1371/journal.pone.0129945.
